# *In vivo* Confocal Microscopic Evaluation of Corneal Dendritic Cell Density and Subbasal Nerve Parameters in Dry Eye Patients: A Systematic Review and Meta-analysis

**DOI:** 10.3389/fmed.2021.578233

**Published:** 2021-04-07

**Authors:** Jing Xu, Peng Chen, Chaoqun Yu, Yaning Liu, Shaohua Hu, Guohu Di

**Affiliations:** School of Basic Medicine, Qingdao University, Qingdao, China

**Keywords:** dry eye, dendritic cell density, subbasal nerve parameters, *in vivo* confocal microscopy, meta-analaysis

## Abstract

**Purpose:** To conduct a systematic review and meta-analysis of the available research on evaluating changes in corneal dendritic cell density (CDCD) and the main subbasal nerve parameters (SNPs) on the ocular surface and assessing the diagnostic performance of *in vivo* confocal microscopy in patients with dry eye disease.

**Methods:** A computerized systematic review of literature published in PUBMED, EMBASE, Web of Science, Scopus, and the Cochrane Central Register of Controlled Trials until May 8, 2020 was performed. All statistical analyses were conducted in *RevMan V.5.3* software. The weighted mean differences (WMDs) and standardized mean differences (SMDs) with 95% confidence intervals (CI) between dry eye patients and healthy subjects were presented as results.

**Results:** A total of 11 studies with 755 participants were recruited, and 931 eyes were included in this meta-analysis. However, not all studies reported both CDCD and SNPs. CDCD in the central cornea was higher (WMD = 51.06, 95% CI = 39.42–62.71), while corneal nerve fiber density (CNFD) and corneal nerve fiber length (CNFL) were lower (WMD = −7.96, 95% CI = −12.12 to −3.81; SMD = −2.30, 95%CI = −3.26 to −1.35) in dry eye patients in comparison with the corresponding values in healthy controls (all *p* < 0.00001).

**Conclusion:** Taken together, while CNFD and CNFL were lower in dry eye patients, central CDCD showed a significant increase in these patients in comparison with the corresponding values in healthy controls.

## Introduction

Dry eye disease (DED) is the most common ocular surface disorder, with hundreds of millions of people affected throughout the world. The latest and authoritative definition of DED was proposed by the Tear Film and Ocular Surface Society Dry Eye Workshop II (TFOS DEWS II) in 2017. The TFOS DEWS II defined DED as a multifactorial disease that is characterized by the loss of homeostasis of the tear film with ocular discomfort symptoms that involves various etiological factors, such as tear film instability, hyperosmolarity, ocular surface inflammation, and neurosensory abnormalities (1). Due to population growth and aging, the prevalence of DED is increasing worldwide, and it currently ranges widely from 5 to 50%, depending on the populations assessed (2). DED seemingly occurs more frequently in Asia than in Western countries (2–4), and it has been reported to occur more frequently in the older population and among women (5–7). Corneal nerve alteration and inflammation both play key roles in DED development (8). However, the mechanisms underlying the discomfort and pain caused by inflammation and the nerve damage in the ocular surface in DED remain unclear.

*In vivo* confocal microscopy (IVCM) is a well-designed and non-invasive approach that allows for observation of the ocular surface structure *in vivo* (9). IVCM can be categorized into tandem-scanning confocal microscopy, slit-scanning confocal microscopy, and the newly developed laser-scanning confocal microscopy (10). Using IVCM in clinical assessments, changes in neuromorphic and ocular surface inflammation can be detected and imaged quantitatively (11). The Heidelberg Retinal Tomograph with the Rostock Cornea Module (HRT/RCM) (Heidelberg Engineering, Dossenheim, Germany) is the only commercially available laser-scanning confocal microscope, and is used widely in the diagnosis of DED due to the higher-quality images and the ability to perform serial scanning (10). The differences among previous studies were attributed to the use of various types of IVCM systems. Therefore, in this meta-analysis, we selected studies that used HRT/RCM to evaluate corneal parameters. In comparison with other devices and tests, HRT/RCM allows assessment of the corneal pathology at the cellular level (12). Although changes in the corneal parameters in DED patients have been demonstrated in many studies, conflicting results still exist, especially those pertaining to the density of the subbasal nerve plexus (13). Therefore, this meta-analysis aimed to assess the corneal parameters, mainly the subbasal nerve parameters (SNPs) and corneal dendritic cell density (CDCD), and evaluate the performance of IVCM in diagnosing DED by collecting data from different studies.

## Methods

### Search Strategy

Databases, including Pubmed, Scopus, EMBASE, Web of Science, and Cochrane Central Register of Controlled Trials, were searched up to May 8, 2020. We developed a search strategy based on Pubmed and made the necessary modifications for each database. The following strategy was used in Pubmed: (dry eye OR dry eye syndrome OR dry eye disease OR xerophthalmia OR xeroma OR keratoconjunctivitis sicca OR Sjögren's Syndrome) AND (*in vivo* confocal microscopy OR confocal microscopy OR IVCM).

### Inclusion and Exclusion Criteria

Inclusion criteria were as follows: (1) at least 10 adults with a definite diagnosis of DED in the test group; (2) a healthy population as the control group; (3) reporting central CDCD and/or at least one corneal nerve parameter (corneal nerve fiber density [CNFD], corneal nerve fiber length [CNFL], corneal nerve branch density [CNBD], or tortuosity coefficient [TC]); (4) using HRT/RCM; and (5) published in English. Studies that met any of the following criteria were excluded: (1) inappropriate types of articles, such as review articles, case reports, editorials, conference papers and abstracts, short surveys, or letters; (2) studies including cases of DED and other ocular disorders simultaneously; (3) studies assessing only animals; (4) studies by the same author (studies with more data, or, in cases involving equal data, the most recently published studies were selected); (5) studies with incomplete raw data; or (6) studies reporting interventions on subjects during trials, such as contact lens wearing, surgery, or anti-inflammatory treatments.

### Data Extraction

**Figure 1:**
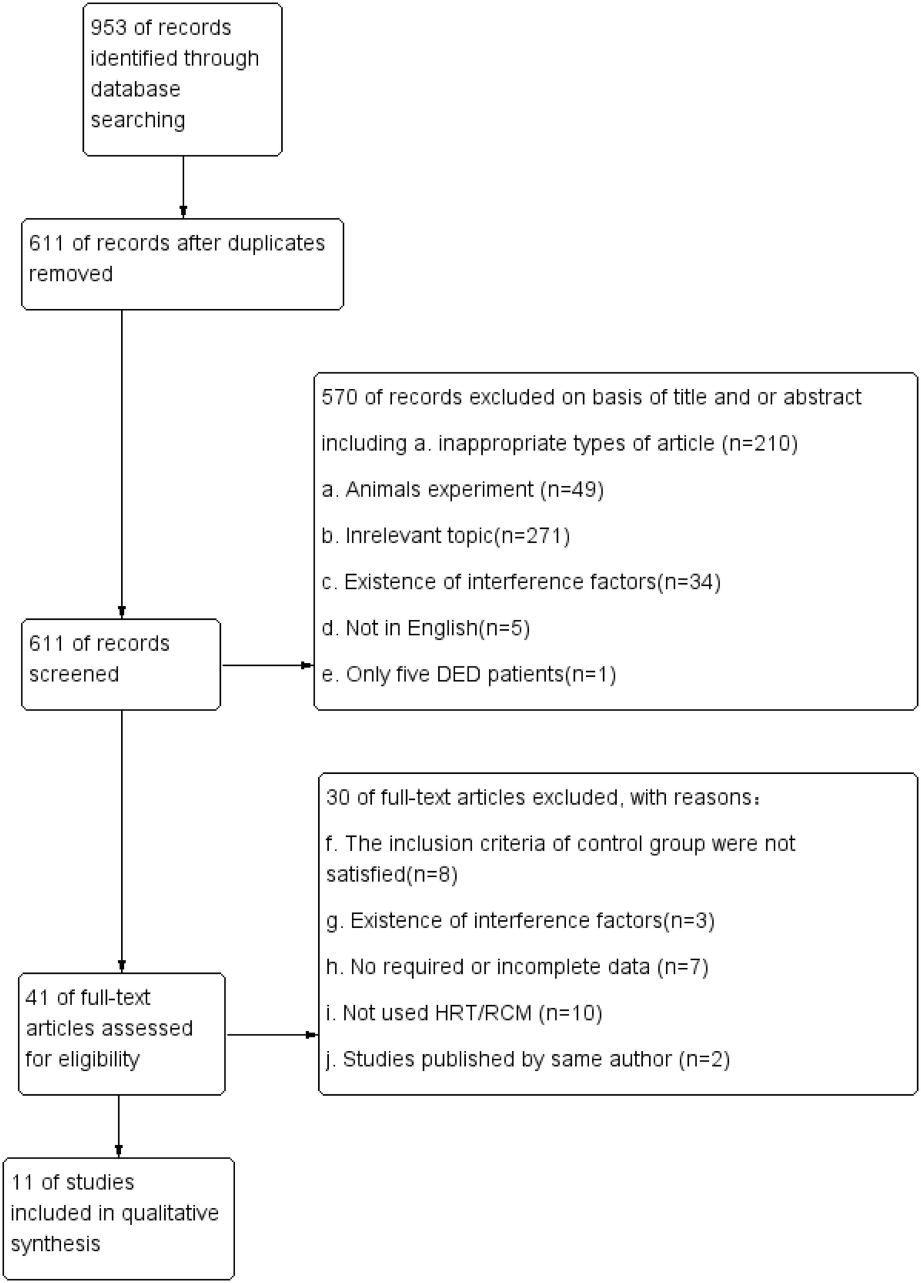
Study flow diagram of article selection.

Before the process of screening, all publications searched were exported to *Endnote* X7. Then, duplicate publications were collated and removed. Two independent reviewers (J.X &Cq.Y) screened eligible titles/abstracts before reading the full article text. Disagreements were resolved via discussion and, if necessary, by consulting a third reviewer (Gh. D). Studies that complied with the inclusion/exclusion criteria were read, and the following information was extracted from the eligible articles: study details (such as the first author's name, year of publication, CDCD, SNPs, and type of IVCM) and patient information (such as mean age, patients' sex, and type of DED). The screening process is summarized in Figure 1, and a-j in the flow diagram describe the screening protocol.

### Assessments of Bias Risks

For this study, we assessed these cross-sectional studies using an 11-item checklist recommended by the Agency for Healthcare Research and Quality (14). Article quality was scored as follows: low quality = 0–3, moderate quality = 4–7, and high quality = 8–11. For case-control studies, the Newcastle-Ottawa Scale was used to rate article quality. This scale assesses studies on three parameters, selection, comparability, and exposure, with a maximum score of nine stars. The studies are rated as follows: low quality = 0–5 stars, medium quality = 6–7 stars, and high quality = 8–9 stars (15).

### Investigation of Heterogeneity

Due to the substantial heterogeneity among the studies, subgroup analysis was conducted to investigate heterogeneity as follows: country of research, type of DED, and IVCM images acquisition and analysis (*post-hoc* analyses that were not pre-planned). Based on the references included, more details were shown in Tables 1–3.

**Table 1:** CDCD in the central cornea by various subgroup meta-analyses.

**Subgroup**	**Group by**	**No of studies**	**Eyes**	**Heterogeneity I^2^(%)**	**WMD of CDCD(cells/mm^2^) (95% CI)**	***p*-value for heterogeneity**
Country of study	Western countries	4	305	62%	51.53 [30.79,72.27]	*P* = 0.05
	Asian countries	3	276	95%	51.41 [34.92, 67.89]	*P* < 0.00001
Type of DED	ADDE	5	314	80%	51.00 [34.82, 67.17]	*P* = 0.0005
	EDE	2	207	0%	43.81 [42.95, 44.67]	*P* = 0.67
Illumination intensity	manual	4	277	38%	49.99 [39.27, 60.71]	*P* = 0.18
	automated	1	147	N	43.80 [42.94, 44.66]	*N*
Number of analyzed images	5	3	244	0%	43.80 [42.94, 44.66]	*P* = 0.85
	3	4	337	83%	56.10 [35.68, 76.53]	*P* = 0.0006
Selecting of analyzed images	randomly	2	107	0%	50.92 [30.01, 71.84]	*P* = 0.83
	subjective judgement	5	474	91%	44.12 [43.27, 44.97]	*P* < 0.00001
Type of counting software	software provided with microscope	4	364	93%	44.10 [43.25, 44.95]	*P* < 0.00001
	Image J	3	217	59%	50.14 [38.32, 61.96]	*P* = 0.09
Location	corneal subbasal plexus	5	424	0.98	3.17 [0.99, 5.36]	*P* < 0.00001
	corneal epithelium	2	157	97%	2.06 [−0.90, 5.02]	*P* < 0.00001

**Table 2:** CNFD in the central subbasal nerve plexus by various subgroup meta-analyses.

**Subgroup**	**Group by**	**No of studies**	**Eyes**	**Heterogeneity I^2^(%)**	**WMD of CNFD(cells/mm^2^) (95% CI)**	***p*-value for heterogeneity**
Country of study	Western countries	4	312	89%	−9.19 [−14.77, −3.62]	*P* < 0.00001
	Asian countries	2	204	97%	−6.04 [−15.40, 3.33]	*P* < 0.00001
Type of DED	ADDE	4	307	83%	−11.60 [−16.63, −6.58]	*P* = 0.0006
	EDE	2	177	88%	−4.91 [−12.67, 2.84]	*P* = 0.004
Number of analyzed images	5	4	320	93%	−5.02 [−8.69, −1.36]	*P* < 0.00001
	3	1	60	N	−13.10 [−17.60, −8.60]	*N*
Selecting of analyzed images	randomly	2	194	81%	−2.38 [−4.69, −0.07]	*P* = 0.02
	subjective judgement	4	322	80%	−11.08 [−15.71, −6.46]	*P* = 0.002
Analysis of images	manual or semi-automated	4	300	91%	−10.79 [−16.65, −4.93]	*P* < 0.00001
	automated	2	216	72%	−2.66 [−5.96, 0.63]	*P* = 0.06

**Table 3:** CNFL in the central subbasal nerve plexus by various subgroup meta-analyses.

**Subgroup**	**Group by**	**No of studies**	**Eyes**	**Heterogeneity I^2^(%)**	**WMD of CNFL (mm/mm^2^) (95% CI)**	***p*-value for heterogeneity**
Country of study	Western countries	4	403	38%	−0.93 [−1.26, −0.61]	*P* = 0.18
	Asian countries	4	315	98%	−6.13 [−10.44, −1.81]	*P* < 0.00001
Type of DED	ADDE	4	321	97%	−1.36 [−3.93, 1.21]	*P* < 0.00001
	EDE	1	147	N	−1.50 [−1.89, −1.10]	*N*
Number of analyzed images	5	5	411	97%	−3.55 [−5.28, −1.83]	*P* < 0.00001
	3	2	198	0%	−1.12 [−1.48, −0.75]	*p* = 0.57
Analysis of images	manual or semi-automated	6	529	96%	−3.05 [−4.47, −1.64]	*P* < 0.00001
	automated	2	216	90%	−1.00 [−1.99, −0.02]	*p* = 0.002

### Statistical Analysis

*Review Manager* V5.3 (RevMan V.5.3) was used for the meta-analysis. We collected the data for continuous variables; the mean, standard deviation, and sample size were extracted from each study. Weighted mean differences (WMDs) with 95% CI values for continuous variable outcomes were calculated for CDCD and CNFD. However, for CNFL, one set of data (16) was about 1,000 times larger than the others. Measurement methods and units of measurement were checked, and no substantial differences were found. We adopted standardized mean differences (SMDs) because of the greatly different data for CNFL. In some studies, the CNFD was defined as total length of corneal nerve fiber (mm/mm^2^), whereas in other studies, it was defined as the number of corneal nerve fibers (n/mm^2^). In order to facilitate comparison, the total corneal nerve length (mm/mm^2^) was considered as CNFL. Meanwhile, the sum of corneal nerves within a frame, in units of n/mm^2^, was considered as CNFD. Heterogeneity of the results of the different studies was tested using the I^2^ value. If *I*^2^ > 50% and *p* < 0.05, significant heterogeneity was indicated statistically. A fixed-effect model was used if *I*^2^ < 50%. Conversely, a random-effects model was applied for significant heterogeneity. Because of the limited number of included studies, bias analysis was not performed.

## Results

### Characteristics of the Eligible Studies

After the screening process, a total of 10 cross-sectional studies (17–25) and one case-control study (26) were included. The 11 studies assessed a total of 755 participants, and 931 eyes met our criteria and were included. The corneal parameters reported by the included studies are shown in Table 4. In the eligible studies, DED patients and healthy controls were matched for age.

**Table 4:** Characteristics of included trials.

**Study**	**Country**	***N***	**Eyes**	**Age (year)**	**Sex (M/F)**	**Group**	**Quality**	**CDCD(n/mm^2^)**	**CNFD (n/mm^2^)**	**CNBD(n/mm^2^)**	**CNFL (mm/mm^2^)**	**TC(rank)**
Cardigos et al. (24)	Portugal	54	54	57.8 ± 11.9	54f	pSS	high		**√**		**√**	
		62	62	60.7 ± 11.0	62f	NSDE						
		20	20	50.9 ± 6.5	20f	Control						
Choi et al. (20)	Korea	44	54	49.3 ± 12.5	19/25	NSDE	moderate				**√**	**√**
		17	34	52.9 ± 22.3	6/11	Control						
Giannaccare et al. (25)	Italy	39	39	64.3 ± 14.5	14/25	DED	moderate		**√**	**√**	**√**	
		30	30	66.1 ± 10.2	12/18	Control						
Kheirkhah et al. (18)	America	45	90	53.7 ± 9.8	17/28	DED	moderate	**√**			**√**	
		15	30	50.7 ± 9.8	7/8	Control						
Kobashi et al. (22)	Japan	25	25	61.8 ± 14.9	3/22	NSDE	moderate	**√**			**√**	
		25	25	61.3 ± 13.6	3/22	Control						
Labbe et al. (16)	China	43	43	46.23 ± 9.74	14/29	NSDE			**√**	**√**	**√**	**√**
		14	14	45.40 ± 9.20	6/8	Control						
Lin et al. (17)	China	14	14	43.8 ± 14.7	1/13	SSDE	moderate	**√**				
		32	32	47.3 ± 14.9	9/23	NSDE						
		33	33	41.8 ± 16.8	16/17	Control						
Nicolle et al. (23)	France	32	32	50.6 ± 3.4	9/23	DED	moderate	**√**	**√**			
		15	15	50.7 ± 7.2	6/9	Control						
Shetty et al. (19)	India	52	104	44.5 ± 40	23/29	EDE	moderate	**√**	**√**	**√**	**√**	
		43	43	41.0 ± 41.48	14/29	Control						
Tepelus et al. (21)	America	22	44	57.5 ± 8.6	1/21	SSDE	moderate	**√**			**√**	**√**
		12	24	58.9 ± 22.4	1/11	NSDE						
		7	10	59.3 ± 12.7	1/6	Control						
Villani et al. (26)	Italy	15	15	52.1 ± 15.4	4/11	SSDE	medium	**√**	**√**			**√**
		15	15	56.3 ± 9.8	5/10	NSDE						
		15	15	55.3 ± 7.3	5/10	MGD						
		15	15	45.2 ± 15.9	5/10	Control						

### Corneal Dendritic Cell Density

**Figure 2:**
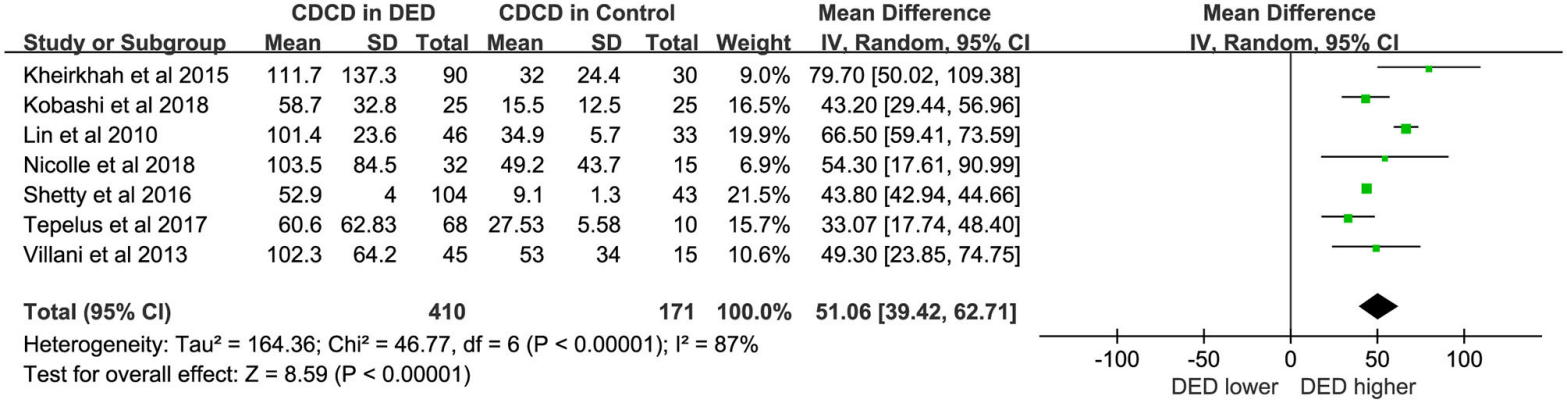
Meta-analysis forest plot of central CDCD in DED patients vs. control group.

Seven studies (17–19, 21–23, 26) with a total of 581 eyes (DED,410; Controls,171) were included in this meta-analysis. As reported by previous studies (18, 19), corneal dendritic cells were identified as bright single dendritic structures with cell bodies. The CDCD in DED patients was significantly higher than that in the controls (WMD = 51.06, 95% CI 39.42–62.71, *p* < 0.00001, *I*^2^ = 87%). Further details were provided in Figure 2.

### Corneal Nerve Fiber Parameters

**Figure 3:**
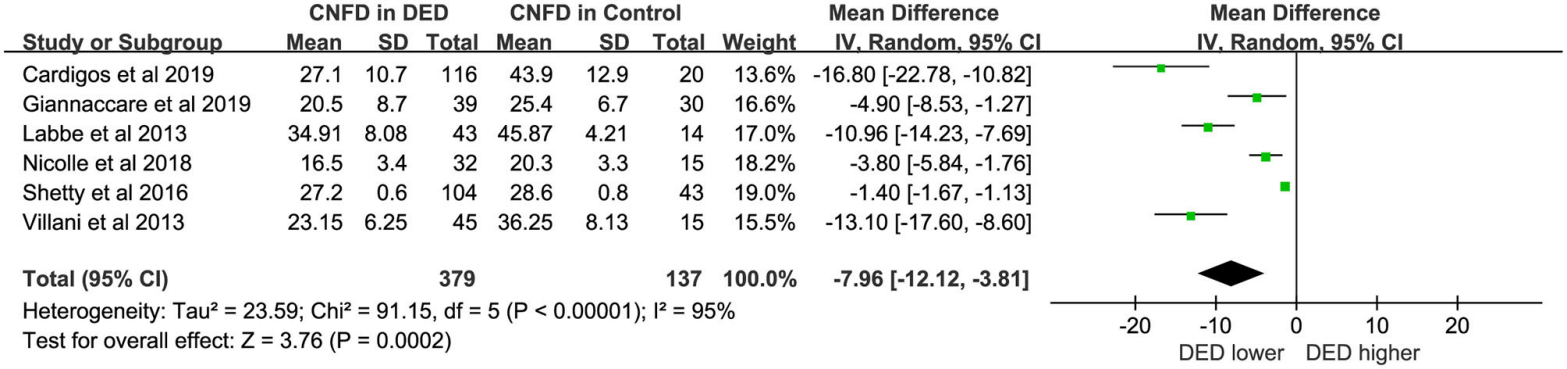
Meta-analysis forest plot of CNFD in DED patients vs. control group.

Six studies (16, 19, 23–26) with 516 eyes (DED, 379; Controls, 137) involved examinations of CNFD using HRT/RCM. The difference in CNFD between DED patients and controls showed statistical significance. The DED group showed lower CNFD than controls (WMD = −7.96, 95% CI −12.12 to −3.81, *p* < 0.00001, *I*^2^ = 95%). For additional details, see Figure 3.

**Figure 4:**
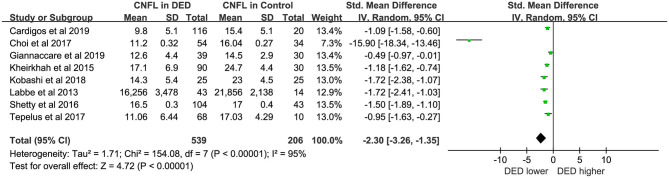
Meta-analysis forest plot of CNFL in DED patients vs. control group.

Eight studies (16, 19, 23–25) with a total of 745 eyes (DED, 539; Controls, 206) were included in the meta-analysis for CNFL. The CNFL in DED was marginally lower than that in healthy controls (SMD = −2.30, 95% CI −3.26 to −1.35, *p* < 0.00001, *I*^2^ = 95%). The detailed results can be found in Figure 4.

### Subgroup Analysis

Due to the high heterogeneity among studies, we performed the following subgroup analyses:

(1) Type of DED: Patients were divided into those with aqueous-deficient dry eye (ADDE) and evaporative dry eye (EDE).
(2) Country: Patients were divided into those from Western and Asian countries.
(3) IVCM images acquisition and analysis: Based on various conditions and different parameters, we did several subgroup analyses.

In assessments based on the type of DED, the high heterogeneity in subgroups for CNFD and CNFL persisted. In subgroup analyses stratified by country, Western countries showed lower heterogeneity than Asian countries for CDCD (*I*^2^ = 62%, *p* = 0.05 vs. *I*^2^ = 95%, *p* < 0.00001), CNFD (*I*^2^ = 91%, *p* < 0.00001 vs. *I*^2^ = 97%, *p* < 0.00001) and CNFL (*I*^2^ = 38%, *p* = 0.18 vs. *I*^2^ = 98%, *p* < 0.00001). However, the overall model showed no significant difference between subgroups in CDCD (*p* = 0.99) and CNFD (*p* = 0.57) without heterogeneity (*I*^2^ = 0%). Conversely, there was a subgroup difference in CNFL (*I*^2^ = 81.9%, *p* = 0.02). The details were shown in Tables 1–3.

For the results of IVCM images' acquisition and analysis, manual illumination intensity might be one of source of heterogeneity (*I*^2^=38%, *p* = 0.18). For analyzing CNFD of images, fully automated analysis might have lower heterogeneity (*I*^2^ = 72%, *p* = 0.06). Of note, due to the small amount of literature, most of the heterogeneity was not reduced. Thus, the results with low heterogeneity seemed unreliable.

### Quality Assessment

The results of the quality assessment were shown in Table 4. Among the studies included, most of the studies were moderate quality, although some unsure risk of bias still exists. In this regard, we performed subgroup analysis. Further details were shown in Tables 1–3.

## Discussion

DED is a typical multifactorial disease with a complex pathophysiology (8). Due to the peculiarity of the cornea, IVCM allows operators to observe corneal nerves and the immune condition in DED and other ocular conditions directly via a non-invasive, quantitative approach. In recent years, growing concern about inflammation and nerve damage has made it important to identify new biomarkers in DED.

### Corneal Dendritic Cell Density

The overall results showed significantly increased CDCD in the central corneal region of DED patients (WMD = 51.06, *P* < 0.00001). However, there was substantial heterogeneity (*I*^2^ > 50%). Therefore, we adopted a random effect model, with subgroup analyses performed to explain heterogeneity. For subgroup analyses, the findings indicated no significant difference (*p* = 0.99) without heterogeneity (*I*^2^ = 0%) between subgroups in different countries. Further details were shown in Figure 2 and Table 1. The nationality of patients cannot be considered a source of heterogeneity. Moreover, another study (26) enrolled DED patients, including those with primary Sjögren's syndrome (pSS), non-Sjögren's syndrome dry eye (NSDE), and meibomian gland diseases (MGDs). Thus, the data for the same control groups were used because MGD belongs to EDE, while pSS and NSDE belong to ADDE. However, as previously reported (18), CDCD in ADDE was significantly higher than that in EDE (*p* = 0.001). In some studies (21, 27), a significant increase was observed in CDCD in Sjögren's syndrome dry eye (SSDE) compared to NSDE. Dendritic cells (DCs) play a key role in pSS (28). Other possible factors may contribute to the strong heterogeneity, such as the definition of DCs, diagnostic criteria of DED, and sex ratio of the subjects. However, due to differences in classifications, we could not evaluate these factors.

In the pathogenesis of dry eye, DCs play an important role in inducing the activation of T cells (29), thus triggering an inflammatory cascade reaction. All the CDCD data included in this study pertained to the central cornea. In one study (17), the data for the center and periphery of the cornea were reported simultaneously. To maintain consistency, we selected the data from the central area of the cornea. The density of corneal epithelial DCs in the periphery and the limbus are reported to be higher than those in the central cornea (30, 31). Animal experiments also confirmed this statement (32). Epithelial DCs are mainly located near the subbasal nerve plexus (11).

### Corneal Nerve Parameters

In short, CNFD and CNFL were lower in DED patients compared with healthy controls (WMD = −7.96, *p* < 0.00001; SMD = −2.30, *p* < 0.00001). And the forest plots of CNFL and CNFD both showed statistically great heterogeneity between studies (Figures 3, 4). However, in the subgroup analysis of different types of DED, the source of this heterogeneity remained elusive (Tables 2, 3). In subgroup analyses, we hypothesized that the pathogenesis of DED patients in Asian countries may be more complicated than that in Western countries. Moreover, in the present study, the exclusion criteria did not include contact lens wearing. Thus, we could not rule out the possibility of DED caused by contact lens wearing. A previous study revealed that wearing contact lenses led to activation and increase in CDCD, as well as a decrease in the subbasal nerve density, indicating that contact lens wear has an impact on the outcome of DED (33).

Only four studies (16, 20, 21, 26) reported corneal nerve TC, and three of those (16, 19, 25) reported CNBD. Therefore, we did not generate the forest plot. Most of the nerve endings that innervate the cornea are located in the SNPs (34). There is no universally agreed definition for corneal nerve parameters, which is one of reasons why changes in corneal nerve parameters varied between different studies. However, there are conflicting results for the difference in CNFD between DED and controls. Some articles (16, 35) have reported a reduction of CNFD in DED, while others reported no difference in SND (36, 37) or a significant increase in corneal nerve density in DED (37, 38). In addition, some studies defined the total length of the nerve fibers per square millimeter as nerve density (18, 20), while others (25, 39, 40) considered the total number of nerves per square millimeter as CNFD. This may be another reason for the conflicting findings. Moreover, variations in nerve density might affect the periods and severity of DED.

### IVCM Images Acquisition and Analysis

For IVCM images acquisition and analysis, although the confocal microscope used in included studies was HRTS, the operating and examination procedure of most studies was subjective. We performed a general quality assessment for the included studies, which indicated IVCM examination might be a major source of risks of bias. The operator selection, image capture, and image analysis were different in different studies. Moreover, the software used to quantify the corneal parameters was not the same. It was reported that CNFL analyzed by manual analysis software (CCMetrics) was higher than using semiautomated or automated software (NeuronJ and ACCMetrics). ACCMetrics was more time-efficient and could provide objective results, since it could distinguish nerve fibers with adjacent pixels by fully automated algorithm (41, 42). Of note, one study (43) has developed a quality evaluation form for the examination of corneal nerve parameters, which is meaningful for future studies. However, the images should be selected randomly to minimize subjective bias. Also, a set of standardized operating procedures should be developed with a unified scanning depth range, a fixed position, and other identical image settings. It might be valuable for future research.

### Limitations and Future Directions

The present study has some limitations that should be considered. There was considerable heterogeneity in the included studies, such as methods for measuring parameters, examined location for the cornea, and ethnic variation. It is probably one factor of the risk of bias in assessment of corneal parameters between dry eye patients and healthy subjects. And for some corneal parameters (e.g., branch density, fiber area), we could not conduct a meta-analysis because of incomplete reporting of data. This meta-analysis might be meaningful in assessing corneal pathology in DED and future relevant research. The quantification of CDCD and corneal nerve parameters by IVCM might be valuable for early diagnosis of dry eye, predicting the severity of dry eye, and contributing to clinical evaluation of anti-inflammatory drug efficacy.

## Conclusion

In summary, the CDCD and SNPs in DED could be examined by IVCM. However, there is still a lack of gold standard criteria for the definitions of parameters and a complete, objective assessment system. In general, CNFD and CNFL were reduced while CDCD showed a significant increase in DED patients. Moreover, IVCM could provide objective markers for diagnosing DED but was not suitable for indicating the subtype of DED.

## Data Availability Statement

The original contributions generated for this study are included in the article/supplementary material, further inquiries can be directed to the corresponding author/s.

## Author Contributions

JX and PC designed the program, searched and reviewed the studies, and were in charge of the manuscript. CY and YL assessed the studies, extracted the data, and wrote the part of the manuscript. SH extracted the data and wrote the part of the manuscript. GD directed the project, contributed to the discussion, reviewed, and edited the manuscript. GD had full access to all the data in the study and had final responsibility for the decision to submit for publication. All authors contributed to the article and approved the submitted version.

## Conflict of Interest

The authors declare that the research was conducted in the absence of any commercial or financial relationships that could be construed as a potential conflict of interest.
